# Validation of the Raw National Aeronautics and Space Administration Task Load Index (NASA-TLX) Questionnaire to Assess Perceived Workload in Patient Monitoring Tasks: Pooled Analysis Study Using Mixed Models

**DOI:** 10.2196/19472

**Published:** 2020-09-07

**Authors:** Sadiq Said, Malgorzata Gozdzik, Tadzio Raoul Roche, Julia Braun, Julian Rössler, Alexander Kaserer, Donat R Spahn, Christoph B Nöthiger, David Werner Tscholl

**Affiliations:** 1 Department of Anesthesiology University Hospital Zurich Zurich Switzerland; 2 Department of Epidemiology and Biostatistics University of Zurich Zurich Switzerland

**Keywords:** workload, questionnaires, National Aeronautics and Space Administration Task Load Index, awareness, situation awareness, patient monitoring, thromboelastometry

## Abstract

**Background:**

Patient monitoring is indispensable in any operating room to follow the patient’s current health state based on measured physiological parameters. Reducing workload helps to free cognitive resources and thus influences human performance, which ultimately improves the quality of care. Among the many methods available to assess perceived workload, the National Aeronautics and Space Administration Task Load Index (NASA-TLX) provides the most widely accepted tool. However, only few studies have investigated the validity of the NASA-TLX in the health care sector.

**Objective:**

This study aimed to validate a modified version of the raw NASA-TLX in patient monitoring tasks by investigating its correspondence with expected lower and higher workload situations and its robustness against nonworkload-related covariates. This defines criterion validity.

**Methods:**

In this pooled analysis, we evaluated raw NASA-TLX scores collected after performing patient monitoring tasks in four different investigator-initiated, computer-based, prospective, multicenter studies. All of them were conducted in three hospitals with a high standard of care in central Europe. In these already published studies, we compared conventional patient monitoring with two newly developed situation awareness–oriented monitoring technologies called Visual Patient and Visual Clot. The participants were resident and staff anesthesia and intensive care physicians, and nurse anesthetists with completed specialization qualification. We analyzed the raw NASA-TLX scores by fitting mixed linear regression models and univariate models with different covariates.

**Results:**

We assessed a total of 1160 raw NASA-TLX questionnaires after performing specific patient monitoring tasks. Good test performance and higher self-rated diagnostic confidence correlated significantly with lower raw NASA-TLX scores and the subscores (all *P*<.001). Staff physicians rated significantly lower workload scores than residents (*P*=.001), whereas nurse anesthetists did not show any difference in the same comparison (*P*=.83). Standardized distraction resulted in higher rated total raw NASA-TLX scores (*P*<.001) and subscores. There was no gender difference regarding perceived workload (*P*=.26). The new visualization technologies Visual Patient and Visual Clot resulted in significantly lower total raw NASA-TLX scores and all subscores, including high self-rated performance, when compared with conventional monitoring (all *P*<.001).

**Conclusions:**

This study validated a modified raw NASA-TLX questionnaire for patient monitoring tasks. The scores obtained correctly represented the assumed influences of the examined covariates on the perceived workload. We reported high criterion validity. The NASA-TLX questionnaire appears to be a reliable tool for measuring subjective workload. Further research should focus on its applicability in a clinical setting.

## Introduction

### Workload

The World Health Organization considers attentive anesthesia providers to be essential to prevent perioperative disability and death [1]. To maintain high quality of care, all factors negatively affecting human performance should be minimized. Various subjective factors, such as high complexity of tasks, stressful personal factors, high-pressure working environment, lack of situation awareness, fatigue, and increased workload, all impair human performance, the quality of care, and thus patient safety [2-4]. The International Organization for Standardization defines workload as the totality of external conditions and requirements in a work system, which affects the physiological and/or psychological state of a person [5]. The perceived workload and a person’s ability to create and maintain adequate situation awareness are interconnected [6]. Situation awareness incorporates the perception of the current status of a situation’s critical elements, with understanding of their meaning, and the projection of this knowledge into the near future [2,7]. For physicians and nurses working inside the operating theatre or intensive care unit, it is crucial to keep situation awareness at a high level through constant mental reassessment. This process however requires substantial cognitive effort. A high workload is a psychological stress factor that takes up part of a person’s naturally limited working memory and ultimately leads to fewer cognitive resources being available.

Hence, accurate assessment of workload is of great importance to manage stressors. Various methods for quantifying perceived workload have been described, which can be divided into the following two large groups: subjective assessment through questionnaires and objective physiological assessment of variables such as heart rate, galvanic skin resistance, breathing rate, pupil diameter, and blinking frequency [8,9].

### National Aeronautics and Space Administration Task Load Index

The National Aeronautics and Space Administration Task Load Index (NASA-TLX) provides the most widely accepted and validated tool to measure overall workload after completing a task [10-12]. It was initially created by the Human Performance Research Group at NASA Ames Research Center for the aviation industry. Since then, its use has expanded to many other fields such as computer science [13], psychophysiology [14], and transportation [15]. The NASA-TLX is a multidimensional tool that contains six predefined dimensions. Three dimensions measure the demands imposed on the subject (mental, physical, and temporal demands), and three dimensions focus on how the subject deals with the task at hand (self-rated performance, effort, and frustration level). Dividing the workload into six subcategories intends to reduce variability among subjects and show the source of the workload. There are two different methods of using the NASA-TLX tool. The weighted NASA-TLX score is a two-step process, in which the user first rates all six subcategories after completing a specific task and then weights the contribution of each factor in a predefined manner. This aims to further understand which potential source accounts mostly for the perceived workload. On the other hand, in the raw NASA-TLX score, the user rates all six subcategories after completing a specific task, without weighing them. The result is the arithmetic mean of all subscales. Research has shown that the raw NASA-TLX has a high correlation with the weighted one [11], but is more time efficient and simpler to apply [16,17]. We used the raw NASA-TLX questionnaire in several studies within the scope of our research activities in the field of patient monitoring and situation awareness–oriented visualization technologies [18].

### Patient Monitoring Tasks

Patient monitoring can be generalized as continuous observation of a condition or certain parameters, regardless of the method used [19,20]. In previous studies, we simplified the presentation of information in different patient monitoring devices by creating two new situation awareness–oriented information transfer technologies called Visual Patient [21,22] and Visual Clot [23]. We further discuss the functionality and applicability of these technologies in the methods section. In tested scenarios, both Visual Patient and Visual Clot helped the health care provider to make correct diagnoses faster and with less perceived cognitive workload compared with standard presentation only.

### Study Aims and Hypotheses

Only few studies have investigated the validity of the raw NASA-TLX score in the health care sector [24-27]. There is no existing gold standard in measuring workload. The primary objective of this study was to validate the raw NASA-TLX questionnaire in patient monitoring tasks by investigating a broad set of over 1000 NASA-TLX scores. We based our evaluation of the robustness of the questionnaire against covariates not associated with workload and on the relation of the NASA-TLX scores with covariates that are known to influence workload. Therefore, we investigated criterion validity. We expect that the scores will increase with the difficulty of the task and distraction, and decrease with the work experience and self-confidence of the user. Further, we assume that the new visualization technologies will reduce all dimensions of the workload approximately uniformly and that not one workload aspect alone will be responsible for observed reductions.

## Methods

### Ethical Permission

The leading ethics committee (Cantonal Ethics Committee of Zurich, Switzerland) reviewed the study protocols for all four included studies and issued a declaration of no objection for each one of them (reference numbers: 2016-00103, 2017-00795, and 2018-00933). Reference number 2017-00795 covers two of the studies, as they were performed simultaneously and included the same participants. Additionally, before participation in this study, we obtained written informed consent from all participants to collect data for scientific purposes and publications.

### Study Design and Data Collection

To validate the NASA-TLX, we analyzed data from four different studies all performed by anesthesia and intensive care providers at the University Hospital Zurich and Cantonal Hospital Winterthur in Switzerland, and University Hospital Frankfurt in Germany [21,23,28,29]. Table 1 provides an overview of the included studies. All of them were investigator-initiated, computer-based, prospective, dual-center studies, and have been published in the past 2 years [21,23,28,29]. In all studies, we based the recruitment of participants on their clinical availability. The day before data collection, we contacted available individuals by institutional email or telephone to plan participation in the study. We then carried out data collection during regular working hours. During this time, we released them from all other duties, including telephone availability.

**Table 1:** Description of the four studies used with the respective participant numbers and completed National Aeronautics and Space Administration Task Load Index questionnaires.

Study title^a^	Location	Number of participants	NASA-TLX^b^ questionnaires, n
Using an Animated Patient Avatar to Improve Perception of Vital Sign Information by Anesthesia Professionals	USZ^c^ and KSW^d^	32	128
Avatar-Based Versus Conventional Vital Sign Display in a Central Monitor for Monitoring Multiple Patients: A Multicenter Computer-Based Laboratory Study	USZ and KSW	38	312
Effects of a Standardized Distraction on Caregivers’ Perceptive Performance with Avatar-Based and Conventional Patient Monitoring: A Multicenter Comparative Study	USZ and KSW	38	312
Improving Decision Making Through Presentation of Viscoelastic Tests as 3D Animated Blood Clot: the Visual Clot	USZ and UKF^e^	60	720

^a^The second and third studies included the same participants.

^b^NASA-TLX: National Aeronautics and Space Administration-Task Load Index.

^c^USZ: University Hospital Zurich.

^d^KSW: Cantonal Hospital of Winterthur.

^e^UKF: University Hospital Frankfurt.

In all included studies, we used the original formulation of the NASA-TLX questionnaire provided by the official NASA website. However, we modified the raw NASA-TLX surveys from six to only five dimensions by removing the physical demand question as our tasks did not require any physical effort. Table 2 shows the modified raw NASA-TLX questionnaire we used in our studies. Participants were staff or resident anesthesiologists and nurse anesthetists with completed specialization qualification. They were all employed in the abovementioned centers during the course of the trials. We selected the participants randomly, regardless of sex, age, job description, staff position, or education level. Participation was voluntary, and none of the subjects received compensation in any form.

**Table 2:** Description of the modified raw National Aeronautics and Space Administration Task Load Index questions and rating scale.

Workload	Descriptive question	Endpoint^a^
Mental demand	Was the task easy or demanding, simple or complex?	0 to 100
Temporal demand	How much time pressure did you feel performing the task?	0 to 100
Self-rated performance^b^	How successful or satisfied did you feel upon the performance or completion of the given task?	0 to 100
Effort	How hard did you have to work (mentally and physically) to accomplish your level of performance?	0 to 100
Frustration level	How insecure, discouraged, stressed, and annoyed versus content, relaxed, and complacent did you feel during the task?	0 to 100

^a^Subjects rated the subscores numerically from 0 (very low) to 100 (very high). The endpoints regarding performance are inverted with 0 indicating very good performance and 100 indicating very poor performance.

^b^The term self-rated performance indicates the performance dimension of the National Aeronautics and Space Administration Task Load Index score.

### Visual Patient and Visual Clot Technologies

**Figure 1:**
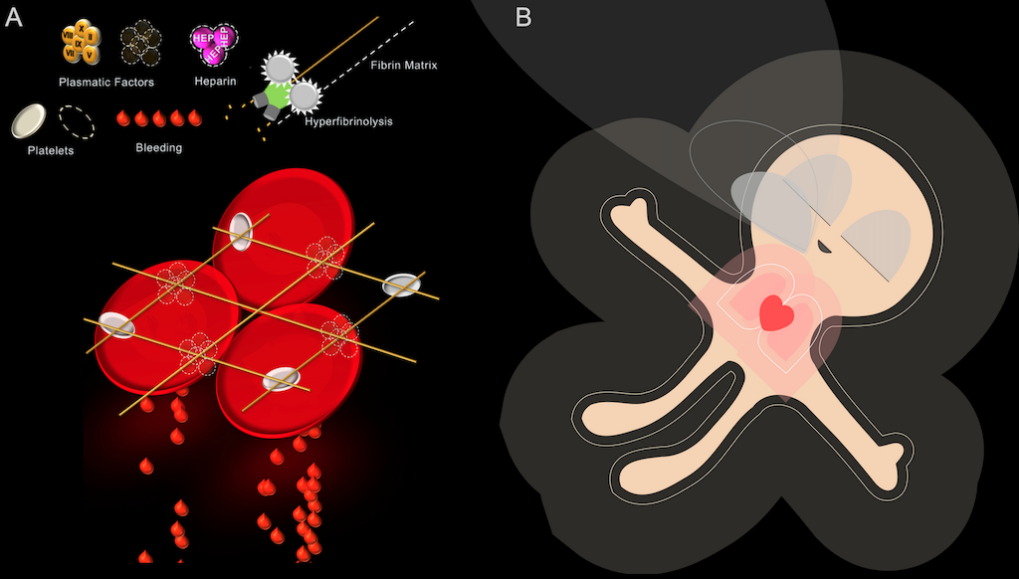
Graphic showing the Visual Clot and Visual Patient technologies. (A) Bleeding Visual Clot illustrated with its different coagulation components. These are either present or absent, depending on the coagulation status. (B) Visual Patient with its different visualizations of vital parameters.

Visual Patient technology is a situation awareness–oriented visualization tool that displays up to 11 of the most frequently used patient vital signs in the form of a patient avatar in addition to the standard monitoring screen [21,22]. Visual Patient transforms the conventional numerical and waveform display of the vital signs in real time into a patient avatar with the ability to adjust its color, shapes, and rhythmic movements depending on the patient’s current situation. For example, if a patient shows an elevated body temperature (ie, more than 37.5°C), the avatar will display heat radiation rising from the patient model. Another example is the patient’s neuromuscular state of relaxation, which is best described by the train-of-four ratio. If the mentioned ratio drops below 20, the patient avatar changes its posture and goes into a floppy state. In situations where several vital signs are out of range, Visual Patient technology is able to illustrate them simultaneously. Visual Clot technology [23] on the other hand illustrates abstract conventional viscoelastic rotational thromboelastometry (ROTEM) readings in form of a three-dimensional animated blood clot. This aims to help the user create a simpler mental model of the current coagulation disorder. The image consists of a blood clot model with its various components such as platelets, coagulation factors, and enzymes. Based on established conventional monitoring values, the visualization shows these coagulation components as either present or not. Figure 1 illustrates both the Visual Clot and Visual Patient technologies. We have provided instructional videos in Multimedia Appendix 1 and Multimedia Appendix 2 explaining how both technologies work.

A relevant limitation for both visualization technologies is the absence of quantification values. Both avatars only display the following three states: too low, within range, and too high. This limitation defines the intended use of the technologies, which is the situation awareness–oriented supplementation of conventional monitoring methods. They were not designed to replace numerical values. Both visualization technologies were invented, prototyped, and patented by our research group at the Institute for Anesthesiology of the University Hospital Zurich. We are developing Visual Patient into a product under a Joint Development and Licensing Agreement with Royal Philips NV and Philips Medizin-Systeme Böblingen GmBH. Regarding Visual Clot, we have signed a letter of intent with Instrumentation Laboratory. Both technologies are in the prototype stage of development and neither Visual Patient nor Visual Clot is currently CE certified as a medical device.

### Patient Monitoring Tasks

We assessed the perceived workload using the raw NASA-TLX questionnaire after performing patient monitoring tasks using either one of the newly developed visualization tools (Visual Patient or Visual Clot) or respective conventional monitoring alone. The monitoring scenarios appeared in randomized order for predefined relatively short periods. Furthermore, the participants had to rate their diagnostic confidence level on a four-point Likert scale ranging from “very unconfident” to “very confident” to further assess uncertainty as a psychological stress factor. In the four mentioned studies, we generated workload through specific tasks as follows:

- In the primary Visual Patient study [21], we displayed four patient monitoring scenarios on a computer for 3 or 10 seconds, portraying either the conventional monitoring screen or the animated patient avatar in randomized order. Subsequently, the participants had to recall the patient’s condition.
- The Visual Patient central monitoring study [29] included four different scenarios in which we showed two critical and four healthy patients simultaneously on a central monitor (as in the intensive care unit or operating room) for 10 or 30 seconds. Afterwards, the participants had to recall the patient’s condition.
- The same participants (as in the central monitoring study [29]) also took part in the Visual Patient distraction study [28]. In this study, we distracted the participants with standardized simple calculation tasks. Simultaneously, we showed them different monitoring scenarios either with the conventional monitoring screen alone or with the help of Visual Patient. The workload task also demanded recall of the patient’s condition.
- Finally, in the Visual Clot study [23], we showed the participants 12 scenarios representing six different hemostatic conditions, which they had to solve using either standard viscoelastic ROTEM results alone or using the matching animated blood clot.

### Validation Method and Studied Covariates

We investigated the robustness of the NASA-TLX questionnaire based on criterion validity, which can be further divided into predictive and concurrent validity. Predictive validity indicates the extent to which an assumption under investigation can be predicted [30]. Concurrent validity compares the result in question with an already known relationship of the same variable [30].

In all included studies, we had consistently recorded 11 different covariates. In this pooled analysis, we determined the expected impact on workload from these measured covariates, using both literature research and logical deductions. It was described that more professional experience should result in lower cognitive workload [31,32]. Since we had recorded the educational stage of the participants through their job descriptions, we regarded this equal to experience. Further, we correlated the measured covariate self-rated confidence with experience and test performance through both literature research and logical reasoning. In order to not confuse the terms, in this manuscript, we defined test performance as the actual testing outcome of the participants and self-rated performance as the subscores of the NASA-TLX. We expected a decreased NASA-TLX score in participants with high self-confidence. Regarding the actual test performance, we investigated predictive validity. We expected good performing participants to perceive less workload. As far as the task’s difficulty is concerned, it cannot be assessed directly as various factors influence it. We defined scenarios in which distraction occurred as more difficult. They divide one’s attention and thus affect the work-related receptiveness [33]. Therefore, we expected the results to show an increased perceived workload and thus correspond to concurrent validity. We did not find any evidence in a literature search and everyday clinical practice of a different perceived workload between the sexes. Therefore, we regarded this as a covariate without an expected influence on workload.

### Statistical Analysis

We validated the raw NASA-TLX score and the different subscores by fitting mixed linear regression models with a random intercept per person (to cover repeated measurements) and a random intercept per study. We fitted univariate models with binary test performance (task correct or incorrect), binary confidence (unconfident or confident), distraction, center, gender, profession, binary daytime (above or below the median), binary playback sequence (first or second half of the tasks), and central monitor performance as covariates.

We analyzed interactions of some covariates with the technology variable to explore more in depth why the NASA-TLX and its subscores improved in the case of the new visual technologies. To explore which subscore benefited the most from the new visualization technologies, we calculated univariate models for each subscore that included only the technology variable, and we compared the size of the estimated coefficients. To characterize the individuals who benefited the most from the introduction of the new technologies, we fitted a joint model for the total NASA-TLX with the technology variable and several other covariates. In one additional model per variable, we included an interaction term between technology and the respective covariate to see if the impact of certain variables was particularly strong in the case of the new technologies.

All analyses were performed using R version 3.6.2 (R Foundation for Statistical Computing). We considered a *P* value <.05 to indicate statistical significance.

### Data Sharing Statement

We provide the complete data used for this study in Multimedia Appendix 3.

## Results

### Study and Participant Characteristics

In all four evaluated studies [21,23,28,29], 128 anesthesia providers participated and rated a total of 1160 NASA-TLX questionnaires. Overall, 552 of 1160 (47.6%) NASA-TLX surveys were collected at the University Hospital Zurich, 360 of 1160 (31.0%) at the University Hospital Frankfurt, and 248 of 1160 (21.4%) at the Cantonal Hospital Winterthur. Further, 648 of 1160 (55.9%) ratings were provided by male participants, and 512 of 1160 (44.1%) by female participants. According to job description, 556 of 1160 (47.9%) ratings were provided by staff physicians, 432 of 1160 (37.2%) by resident physicians, and 172 of 1160 (14.8%) by nurse anesthetists. Comparing the technologies used, 62.1% (720/1160) of all data originated from the three Visual Patient projects, and 37.9% (440/1160) from the Visual Clot study.

### Quantitative Analyses of the NASA-TLX Questionnaire

**Figure 2:**
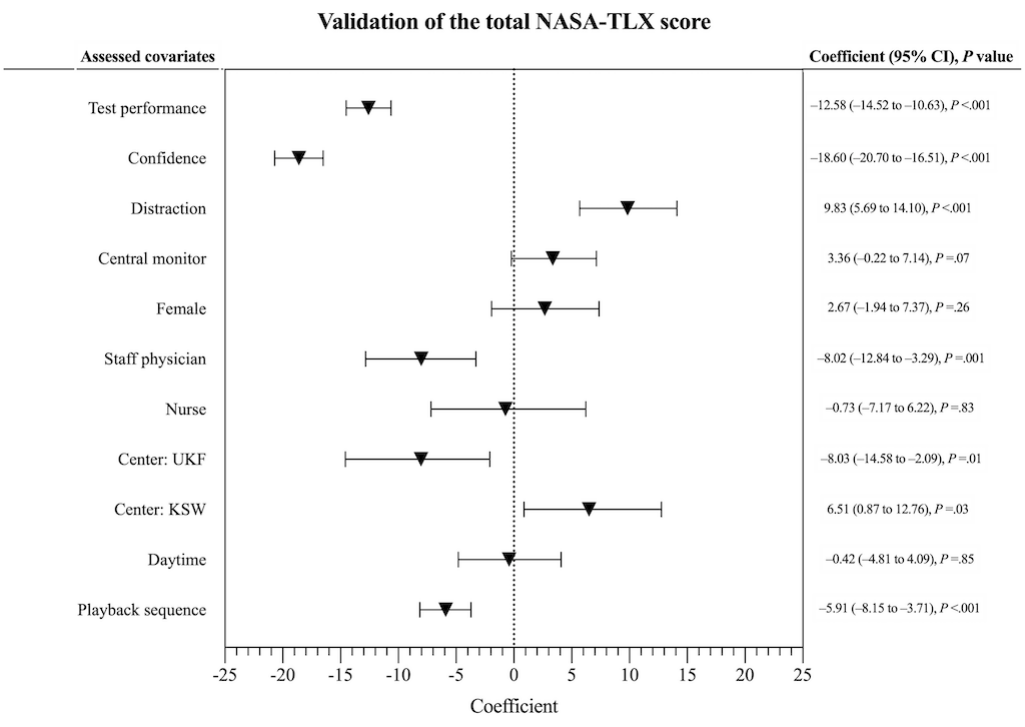
Correlation of different covariates with the total score of the National Aeronautics and Space Administration Task Load Index (NASA-TLX) workload assessment tool. Left and right of the dashed line indicate lower and higher perceived workload, respectively. KSW: Cantonal Hospital Winterthur; UKF: University Hospital Frankfurt.

**Figure 3:**
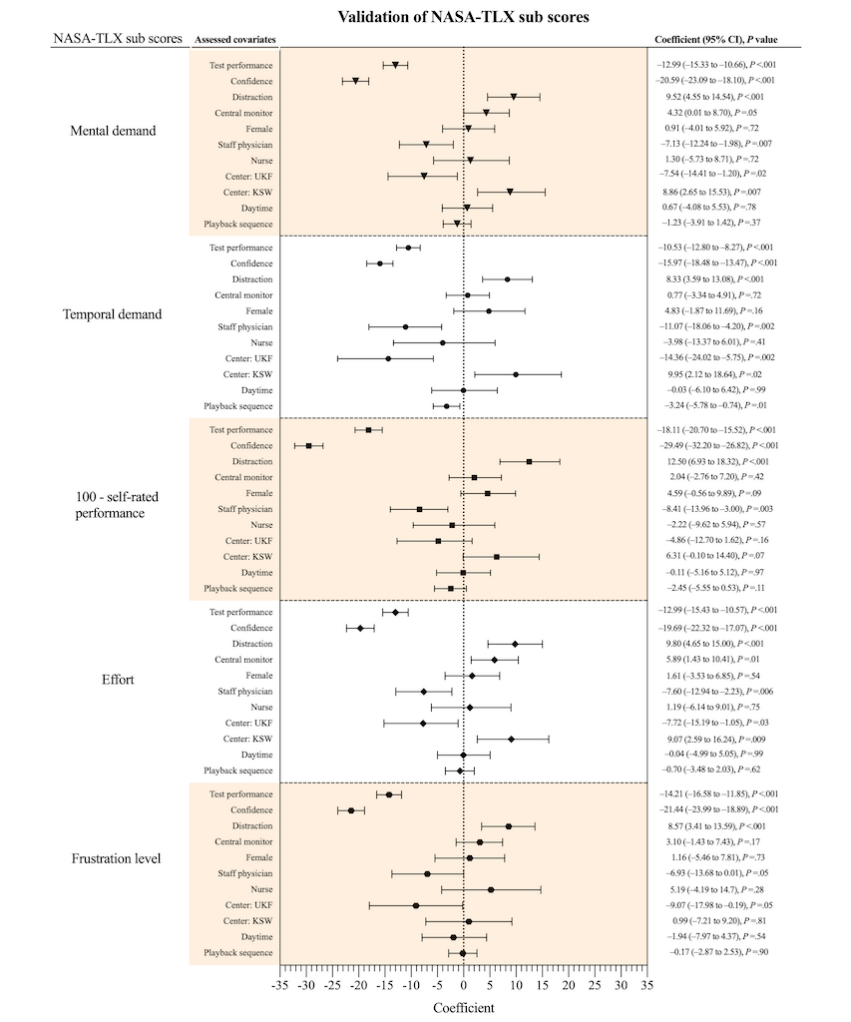
Correlation of different covariates with subscores of the National Aeronautics and Space Administration Task Load Index (NASA-TLX) workload assessment tool. The original NASA-TLX questionnaire evaluated performance on an inverted X-axis from perfect to failure, with a low raw score corresponding to good self-rated performance. Therefore, to ensure that the X-axis in this figure is the same, the self-rated performance is displayed inverted. KSW: Cantonal Hospital Winterthur; UKF: University Hospital Frankfurt.

In Figure 2 and Figure 3, we illustrate the correlation of the total NASA-TLX workload score and its subscores after performing univariate analysis with test performance, confidence, distraction, center, gender, job description, daytime, and playback sequence as different covariates. The playback sequence was described as the first or second half of the task. We provide the NASA-TLX coefficient and the 95% CI.

The total workload score analysis showed that participants’ test performance and higher confidence correlated significantly with lower NASA-TLX scores (both *P*<.001). Compared with the University Hospital Zurich, participants from the University Hospital Frankfurt had significantly lower total workload scores (−8.03, 95% CI −14.58 to −2.09; *P*=.01). Further, the second half of the playback sequence had a significant positive effect on lowering the perceived workload (−5.91, 95% CI −8.15 to −3.71; *P*<.001). Regarding the job position, resident physicians served as the comparison. Staff physicians (−8.02, 95% CI −12.84 to −3.29; *P*=.001) rated significantly lower workload than residents, whereas nurses did not show any significant difference compared with residents (*P*=.83). Distraction and the Cantonal Hospital Winterthur compared with the University Hospital Zurich correlated significantly with higher rated NASA-TLX scores (*P*<.001 and *P*=.03, respectively). The other listed covariates did not show any relevant difference.

Figure 3 illustrates the entire evaluation of the examined covariates for the subscores of the NASA-TLX. Good test performance and high confidence level after performing the task correlated significantly with lower workload scores in every subcategory of the NASA-TLX (all *P*<.001). Staff physicians also differed significantly from resident physicians with lower workload scores in every subcategory, except the frustration level (−6.93, 95% CI −13.68 to 0.01; *P*=.05). Distraction was the only covariate to show a relevant effect on increasing the workload in every subscore of the NASA-TLX. Comparing the participants at the different centers with those at the University Hospital Zurich, the participants at the University Hospital Frankfurt had less mental (*P*=.02) and temporal (*P*=.002) demands, and less perceived effort (*P*=.003). The participants at the Cantonal Hospital Winterthur had more mental (*P*=.007) and temporal demands (*P*=.02), and more required effort (*P*=.03). We observed no gender difference in all subscores. Showing several patients simultaneously on a central monitor correlated significantly with increased mental demand (*P*=.05) and increased effort (*P*=.01) to fulfill the task. We observed no effect for temporal demand (0.77, 95% CI −3.34 to 4.91; *P*=.72), frustration level (3.10, 95% CI −1.43 to 7.43; *P*=.17), and self-rated performance (2.04, 95% CI −2.76 to 7.20; *P*=.42) using a central monitor. Playback sequence showed its only significant effect on the temporal demand subscore with lower perceived workload in the second half of the task (−3.24, 95% CI −5.78 to −0.74; *P*=.01).

Performing the same tasks, we compared the conventional monitoring devices with the respective visualization technologies. The total NASA-TLX score and all listed subscores correlated significantly with lower workload scores when using Visual Patient and Visual Clot (all *P*<.001). Table 3 demonstrates the comparison of the new visualization technologies with respective conventional monitoring. The self-rated performance dimension showed the most relevant change in the perceived workload assessment (coefficient −18.28).

**Table 3:** Overall comparison of the new visual technologies Visual Patient and Visual Clot with conventional monitoring.

Variable	Coefficient	CI lower	CI upper	*P* value
Total	−14.36	−16.06	−12.65	<.001
Mental demand	−15.79	−17.85	−13.72	<.001
Temporal demand	−10.53	−12.59	−8.46	<.001
Self-rated performance^a^	−18.28	−20.63	−15.93	<.001
Effort	−16.80	−18.92	−14.68	<.001
Frustration level	−15.97	−18.06	−13.87	<.001

^a^The term self-rated performance indicates the performance dimension of the National Aeronautics and Space Administration Task Load Index score.

### Data Sharing Statement

We provide the complete analysis of used data for this study in Multimedia Appendix 4.

## Discussion

### Principal Findings

This pooled analysis examined a broad set of raw NASA-TLX scores obtained after performing patient monitoring tasks. Good test performance, high self-confidence in successfully completing a task, high hierarchical job position, and training correlated with decreased raw NASA-TLX workload scores, whereas distraction scenarios increased perceived workload. The questionnaire was robust against nonworkload-related factors such as gender. The new visualization technologies Visual Patient and Visual Clot both decreased all workload dimensions compared with conventional monitoring alone and patient monitoring reference values provided by the literature [11]. Reducing workload helps to free cognitive resources, which can be used to understand the provided monitoring information crucial to maintain a high quality of care [7].

The analysis of 1160 raw NASA-TLX questionnaires showed that self-rated high confidence correlated significantly with lower overall raw NASA-TLX scores and its subscores (all *P*<.001). Furthermore, staff physicians had significantly lower total workload scores compared with residents. During clinical residency training, inexperienced physicians go through a period of professional and personal growth. They acquire knowledge and skills [6], and experience many challenging clinical situations that build professional competency and thereby increase their confidence at work [34]. Generally, staff physicians are more certain of their clinical abilities and thus exude more confidence than residents. The participants at the University Hospital Frankfurt also rated lower total workload scores compared with those at the University Hospital Zurich. This is in line with our abovementioned train of thought, as the participating physicians in Frankfurt had more professional experience overall than those in Zurich [23]. When comparing nurses with residents, there was no difference in the perceived total workload after performing the same monitoring task. We explain this interesting finding on the basis of existing practical experience. All nurses who took part in this study had completed their specialty qualification and thus often had more professional experience than the residents who were still in training. Their self-confidence may have increased owing to their completed education. Our results confirm our hypothesis that confidence as a trait, which is compatible with a higher job position, lowers perceived workload. It is known from other domains that individuals with higher confidence make better decisions [35]. The World Health Organization aims to continuously improve, protect, and promote the health, safety, and well-being of all workers [36]. Analyzing workload in the perioperative setting can lead directly to practical work-based implications, such as tailored task assignment and improved training plans, for residents or other personnel. This efficient identification and management of workload influences employee well-being [37] and lowers the source of fatigue from work overload, which is an independent risk factor for exhaustion and burnout [38]. Moreover, workload has been shown to be associated with adverse patient outcomes [39-41].

Good test performance in conducting patient monitoring tasks correlated with lower total raw NASA-TLX scores, as well as all subscores, including high self-rated performance. We propose that this connection resulted from both training and experience. When a person has received a lot of training and thus experience in performing a task, the perceived workload decreases and the actual performance increases. Nevertheless, this finding allows not drawing a linear relationship between task performance and workload. This relation is complex when investigated in more detail. Both excessive workload and low demand situations can degrade performance [42], and additional factors, such as personal resilience, influence the work capacity to a great extent. This shows that there are other factors apart from workload that influence task performance.

Further, our results showed that standardized distraction negatively affected the raw NASA-TLX scores with all subcategories. This is in line with our hypothesis that distractions increase perceived workload. They take up part of the already limited mind while coping with several things simultaneously and reduce the work-related memory capacity of humans. It was shown that distractions impair situational awareness and thus affect clinical decision making [33,43]. The source of most anesthesia adverse events lies in reduced situational awareness [4,44]. This further demonstrates the importance of minimizing working environment distractions in areas that involve workload-sensitive tasks such as patient monitoring inside the operating theatre [45]. In one study, 22 of 25 (88%) anesthesia providers agreed to the statement that human factor problems do lead to critical information not being received [46].

The newly developed visualization technologies Visual Patient and Visual Clot were associated with decreased perceived workload as expected. These technologies have been developed to link several sources of information together and create an avatar-based visualization aimed to facilitate the mental model of the current situation [21-23]. Endsley defined the goal of optimal situation awareness–oriented design to transfer information as quickly as possible and with the least cognitive effort [7].

Decreasing workload while monitoring a patient reduces psychological stress by saving cognitive resources, which are especially needed in critical situations. In 2015, Grier et al [11] examined a vast amount of published NASA-TLX scores in a meta-analysis showing the distribution frequency of the scores by task type. They analyzed 174 monitoring-type tasks involving change detection, speech detection, and vigilance tasks. A mean NASA-TLX global workload score of 52.24 with an IQR of 22.66 (39.97-62.63) was reported [11]. This fits the mean NASA-TLX global workload score of 54.60 with an IQR of 27.0 (42.0-69.0), which we observed when evaluating our conventional monitoring tasks. Using the situation awareness–oriented visualization monitoring technologies, we found a mean NASA-TLX global workload score of 40.2 with an IQR of 31.0 (25.0-56.0). These technologies lowered the mean perceived workload compared with the literature value by 23.0%. This is comparable to a workload that occurs when driving a car (mean 41.52, IQR 23.7 [28.05-51.73]) [11].

This study contributes to the ongoing validation of the NASA-TLX in the medical field. The results are consistent with those of other studies that have found high construct validity of the NASA-TLX score [24,25]. These studies described an increase in the workload score due to distraction [47], case complexity [24], and low task performance [48], and a reduction due to training [49]. Other studies found good correlation with other questionnaires for workload assessment [50,51] and with physiological stress measurements [52,53].

### Strengths and Limitations

Our study had several limitations. All tasks used to validate the raw NASA-TLX questionnaire took place in a testing environment with clearly defined monitoring limits within scenarios. Perceived workload and therefore its assessment might differ in a clinical setting, where each situation must be interpreted independently. Future studies should test the applicability of the raw NASA-TLX in the clinical setting. However, it is plausible that this effect is marginal as the score reflects a subjective evaluation while information intake and thus perception of workload remain similar. Further, we assessed and validated a modified version of the questionnaire. Since our monitoring tasks did not require any relevant physical effort, we removed this dimension from the scale in order not to distort the value of the total workload assessed. Future studies are required to investigate whether such a modification affects the internal consistency of the NASA-TLX questionnaire. Moreover, a true validation would correlate obtained scores with objectively measurable stress characteristics such as heart rate and pupil diameter. Another limitation is that all patient monitoring scenarios took place in central Europe in high quality of care hospitals. Perceived workload can differ in other parts of the world and might influence the reproducibility of the assessment. Finally, interpretation of the raw NASA-TLX scores requires comparative values after performing similar tasks. Therefore, more available data using the same questionnaire in patient monitoring tasks would reduce this limitation. Nevertheless, the results of this study support the use of the raw NASA-TLX score in patient monitoring tasks.

Among the particular strengths of this study are the multicenter design, the large data set, and the consistent recording of identical covariates in all included studies. We examined more than 1000 raw NASA-TLX questionnaires, which were completed by 130 participants, with individual selection solely based on daily clinical availability. This large proportion of staff from the respective institutions constitutes a representative sample. Further, in all included studies, intraparticipant comparisons took place as there was an evaluation of the same monitoring task with both examined interface designs (ie, conventional versus visualization). This greatly reduces the influence of confounding variables if the main factor responsible for the difference remains the interface modality shown, and it further increases the quality of the study.

### Conclusions

For patient monitoring, this study validated a modified version of the raw NASA-TLX questionnaire, in which the physical dimension had been removed from the scale owing to the nature of the given tasks. The obtained scores correctly depicted the assumed influences of the covariables that affect perceived workload. This provided a high extent of criterion validity. The modified raw NASA-TLX questionnaire appears to be a reliable tool for measuring the subjective workload of anesthesia providers who monitor patients inside the operating room. Further research is needed to investigate the applicability of the NASA-TLX questionnaire in the clinical setting and its transferability to personnel working in intensive care units. Moreover, a true validation study for the subjective workload assessment should correlate the NASA-TLX scores with objectively measurable stress characteristics such as heart rate and pupil diameter.

## Appendix

Multimedia Appendix 1 Instructional video explaining the Visual Clot technology.

Multimedia Appendix 2 Instructional video explaining the Visual Patient technology.

Multimedia Appendix 3 Complete data for this study.

Multimedia Appendix 4 Complete analysis of used data for this study.

## Abbreviations

NASA-TLX: National Aeronautics and Space Administration-Task Load Index
ROTEM: rotational thromboelastometry
