# Supplementary material for: Validation of the Raw National Aeronautics and Space Administration Task Load Index (NASA-TLX) Questionnaire to Assess Perceived Workload in Patient Monitoring Tasks: Pooled Analysis Study Using Mixed Models
Source: J Med Internet Res. 2020 Sep 7;22(9):e19472. doi: 10.2196/19472 (PMC7506540; doi:10.2196/19472)
Supplement: Multimedia Appendix 4 [file jmir_v22i9e19472_app4.pdf]

Abbreviations used in this document:

- NASA-TLX	= NASA Task Load Index
- VC	= Visual Clot
- VP	= Visual Patient
- ROTEM	= Rotational Thromboelastometry
- USZ	= University Hospital Zurich (Switzerland)
- KSW	= Cantonal Hospital Winterthur (Switzerland)
- UKF	= University Hospital Frankfurt (Germany)
- Conv	= Conventional monitoring
- CM	= Central monitor

NASA-TLX Validation Study

Version 4.0
Julia Braun

April 14, 2020

Changes in comparison to the previous version:

- Addition of variable ”performance reversed” to Table 19
- Additional descriptive table of joint data set

The aims of this study are:

1. Validate NASA-TLX
2. Explore interactions of some covariates with the technology variable

1 Descriptives

We first show descriptive tables (one for the continuous and one for the categorical variables) for each separate study and for the joint data set. ROTEM = Rotational thromboelastometry

1.1 VC

Variable	n	Min	q_1	$\widetilde{x}$	$\bar{x}$	q_3	Max	s	IQR	#NA
min_past_7am	714	78	226.0	304.0	333.4	463.0	630	133.0	237.0	6
Experience_years	720	0	4.0	7.5	8.2	11.0	29	5.8	7.0	0
ROTEM_per_year	720	0	11.5	40.0	38.7	53.0	100	30.3	41.5	0
Self_rated_ROTEM_skill	720	0	25.5	50.0	46.6	70.0	90	26.3	44.5	0
time.to.decision	698	4	12.0	20.0	25.9	34.0	139	19.5	22.0	22
NASA_TLX	698	0	23.2	41.0	40.5	56.0	95	21.5	32.8	22
NASA_TLX_Mental_demand	698	0	23.0	41.0	42.4	63.0	100	24.9	40.0	22
NASA_TLX_Temporal_demand	698	0	19.0	38.0	39.9	60.0	100	25.6	41.0	22
Reverse_NASA_TLX_Overall	698	0	19.0	34.0	37.9	58.8	100	25.6	39.8	22
NASA_TLX_Effort	698	0	22.0	43.0	43.0	62.8	100	25.3	40.8	22
NASA_TLX_Frustration	697	0	19.0	36.0	38.3	58.0	100	24.2	39.0	23

Table 1: Descriptive table - continuous data: Visual Clot (VC) study

Variable	Levels	n	%
Center	USZ	360	50.0
	UKF	360	50.0
	KSW	0	0.0
	all	720	100.0
Median_past_7am	No	402	55.8
	Yes	318	44.2

	all	720	100.0
Sex	male	444	61.7
	female	276	38.3
	all	720	100.0
Job	Resident	300	41.7
	Staff Physician	420	58.3
	Nurse	0	0.0
	all	720	100.0
Playback_binary	First two	120	16.7
	Later	600	83.3
	all	720	100.0
Technology	ROTEM/Conv	349	50.0
	VC/VP	349	50.0
	all	698	100.0
Scenario	1	48	6.9
	2	72	10.3
	3	48	6.9
	4	72	10.3
	5	72	10.3
	6	36	5.2
	7	48	6.9
	8	72	10.3
	9	120	17.2
	10	38	5.4
	11	72	10.3
	all	698	100.0
Distraction	No	703	100.0
	Yes	0	0.0
	all	703	100.0
confidence_category	Very unconfident	36	5.2
	Unconfident	148	21.2
	Confident	276	39.5
	Very confident	238	34.1
	all	698	100.0
confidence_binary	Below median	189	26.9
	Above median	514	73.1
	all	703	100.0

Table 2: Descriptive table - categorical data: VC study

1.2 VP1

Variable	n	Min	q_1	$\widetilde{x}$	$\bar{x}$	q_3	Max	s	IQR	#NA
min_past_7am	128	143	381.2	486.5	452.3	543.8	612	118.1	162.5	0
Experience_years	NA	NA	NA	NA	NA	NA	NA	NA	NA	NA
ROTEM_per_year	NA	NA	NA	NA	NA	NA	NA	NA	NA	NA
Self_rated_ROTEM_skill	NA	NA	NA	NA	NA	NA	NA	NA	NA	NA
time.to.decision	NA	NA	NA	NA	NA	NA	NA	NA	NA	NA
NASA_TLX	128	26	37.0	46.0	51.3	66.0	93	16.5	29.0	0
NASA_TLX_Mental_demand	128	20	65.0	76.0	75.7	90.0	100	17.8	25.0	0
NASA_TLX_Temporal_demand	128	16	64.0	83.0	77.3	95.0	100	21.3	31.0	0
Reverse_NASA_TLX_Overall	128	0	20.8	49.0	47.1	71.2	98	28.7	50.5	0
NASA_TLX_Effort	128	17	60.0	75.0	73.0	90.2	100	20.8	30.2	0
NASA_TLX_Frustration	128	0	33.2	51.0	53.4	72.0	100	26.1	38.8	0

Table 3: Descriptive table - continuous data: VP1 study

Variable	Levels	n	%
Center	USZ	64	50.0
	UKF	0	0.0
	KSW	64	50.0
	all	128	100.0
Median_past_7am	No	24	18.8
	Yes	104	81.2
	all	128	100.0
Sex	male	60	46.9
	female	68	53.1
	all	128	100.0
Job	Resident	28	21.9
	Staff Physician	48	37.5
	Nurse	52	40.6
	all	128	100.0
Playback_binary	First two	64	50.0
	Later	64	50.0
	all	128	100.0
Technology	ROTEM/Conv	64	50.0
	VC/VP	64	50.0
	all	128	100.0
Scenario	12	32	25.0
	13	32	25.0
	14	32	25.0
	15	32	25.0
	all	128	100.0
Distraction	No	128	100.0
	Yes	0	0.0
	all	128	100.0
confidence_category	Very unconfident	27	21.1
	Unconfident	19	14.8
	Confident	37	28.9
	Very confident	45	35.2

	all	128	100.0
confidence_binary	Below median	48	37.5
	Above median	80	62.5
	all	128	100.0

Table 4: Descriptive table - categorical data: VP1 study

1.3 VP2

Variable	n	Min	q_1	$\tilde{x}$	$\bar{x}$	q_3	Max	s	IQR	#NA
min_past_7am	312	42	200.0	316	314.9	464	570	157.2	264.0	4
Experience_years	NA	NA	NA	NA	NA	NA	NA	NA	NA	NA
ROTEM_per_year	NA	NA	NA	NA	NA	NA	NA	NA	NA	NA
Self_rated_ROTEM_skill	NA	NA	NA	NA	NA	NA	NA	NA	NA	NA
time.to.decision	NA	NA	NA	NA	NA	NA	NA	NA	NA	NA
NASA_TLX	312	14	48.8	62	61.3	75	100	19.0	26.2	4
NASA_TLX_Mental_demand	312	10	70.0	80	76.7	90	100	20.0	20.0	4
NASA_TLX_Temporal_demand	312	0	60.0	80	71.7	90	100	27.5	30.0	4
Reverse_NASA_TLX_Overall	312	0	30.0	50	52.0	70	100	24.3	40.0	4
NASA_TLX_Effort	312	10	60.0	80	73.1	90	100	21.4	30.0	4
NASA_TLX_Frustration	312	0	5.0	30	32.8	50	100	30.0	45.0	4

Table 5: Descriptive table - continuous data: VP2 study

Variable	Levels	n	%
Center	USZ	128	41.0
	UKF	0	0.0
	KSW	184	59.0
	all	312	100.0
Median_past_7am	No	152	48.7
	Yes	160	51.3
	all	312	100.0
Sex	male	144	46.1
	female	168	53.9
	all	312	100.0
Job	Resident	104	33.3
	Staff Physician	88	28.2
	Nurse	120	38.5
	all	312	100.0
Playback_binary	First two	156	50.0
	Later	156	50.0
	all	312	100.0
Technology	ROTEM/Conv	156	50.0
	VC/VP	156	50.0
	all	312	100.0
Scenario	16	92	29.5
	17	64	20.5
	18	46	14.7
	19	46	14.7
	20	32	10.3
	21	32	10.3
	all	312	100.0
Distraction	No	234	75.0
	Yes	78	25.0
	all	312	100.0
confidence_binary	Below median	115	36.9
	Above median	197	63.1

all	312	100.0

Table 6: Descriptive table - categorical data: VP2 study

1.4 Total

Variable	n	Min	q_1	$\tilde{x}$	$\bar{x}$	q_3	Max	s	IQR	#NA
min_past_7am	1154	42	225.0	316.0	341.6	467.0	630	144.0	242.0	10
Experience_years	720	0	4.0	7.5	8.2	11.0	29	5.8	7.0	444
ROTEM_per_year	720	0	11.5	40.0	38.7	53.0	100	30.3	41.5	444
Self_rated_ROTEM_skill	720	0	25.5	50.0	46.6	70.0	90	26.3	44.5	444
time.to.decision	698	4	12.0	20.0	25.9	34.0	139	19.5	22.0	466
NASA_TLX	1138	0	32.2	48.0	47.4	64.0	100	22.3	31.8	26
NASA_TLX_Mental_demand	1138	0	32.0	60.0	55.6	80.0	100	28.3	48.0	26
NASA_TLX_Temporal_demand	1138	0	27.0	52.0	52.8	80.0	100	30.5	53.0	26
Reverse_NASA_TLX_Overall	1138	0	23.0	40.0	42.8	63.8	100	26.4	40.8	26
NASA_TLX_Effort	1138	0	32.0	58.0	54.6	77.8	100	27.9	45.8	26
NASA_TLX_Frustration	1137	0	18.0	36.0	38.5	60.0	100	26.7	42.0	27

Table 7: Descriptive table - continuous data: joint data set

Variable	Levels	n	%
Center	USZ	552	47.6
	UKF	360	31.0
	KSW	248	21.4
	all	1160	100.0
Median_past_7am	No	578	49.8
	Yes	582	50.2
	all	1160	100.0
Sex	male	648	55.9
	female	512	44.1
	all	1160	100.0
Job	Resident	432	37.2
	Staff Physician	556	47.9
	Nurse	172	14.8
	all	1160	100.0
Playback_binary	First two	340	29.3
	Later	820	70.7
	all	1160	100.0
Technology	ROTEM/Conv	569	50.0
	VC/VP	569	50.0
	all	1138	100.0
Scenario	1	48	4.2
	2	72	6.3
	3	48	4.2
	4	72	6.3
	5	72	6.3
	6	36	3.2
	7	48	4.2
	8	72	6.3
	9	120	10.5
	10	38	3.3
	11	72	6.3
	12	32	2.8
	13	32	2.8

	14	32	2.8
	15	32	2.8
	16	92	8.1
	17	64	5.6
	18	46	4.0
	19	46	4.0
	20	32	2.8
	21	32	2.8
	all	1138	100.0
Distraction	No	1065	93.2
	Yes	78	6.8
	all	1143	100.0
confidence_category	Very unconfident	63	7.6
	Unconfident	167	20.2
	Confident	313	37.9
	Very confident	283	34.3
	all	826	100.0
confidence_binary	Below median	352	30.8
	Above median	791	69.2
	all	1143	100.0

Table 8: Descriptive table - categorical data: joint data set

**Variable**	**Levels**	**n**	**Min**	q_1	$\tilde{x}$	$\bar{x}$	q_3	**Max**	**s**	**IQR**	**#NA**
NASA_TLX	ROTEM/Conv	569	1	42.0	56.0	54.6	69.0	100	20.3	27.0	0
	VC/VP	569	0	25.0	38.0	40.2	56.0	98	21.9	31.0	0
	all	1138	0	32.2	48.0	47.4	64.0	100	22.3	31.8	26
NASA_TLX_Mental_demand	ROTEM/Conv	569	1	49.0	67.0	63.5	80.0	100	24.8	31.0	0
	VC/VP	569	0	24.0	47.0	47.7	74.0	100	29.3	50.0	0
	all	1138	0	32.0	60.0	55.6	80.0	100	28.3	48.0	26
NASA_TLX_Temporal_demand	ROTEM/Conv	569	0	35.0	61.0	58.1	80.0	100	29.3	45.0	0
	VC/VP	569	0	20.0	46.0	47.5	75.0	100	30.8	55.0	0
	all	1138	0	27.0	52.0	52.8	80.0	100	30.5	53.0	26
Reverse_NASA_TLX_Overall	ROTEM/Conv	569	0	31.0	51.0	51.9	71.0	100	25.7	40.0	0
	VC/VP	569	0	17.0	30.0	33.6	50.0	100	23.8	33.0	0
	all	1138	0	23.0	40.0	42.8	63.8	100	26.4	40.8	26
NASA_TLX_Effort	ROTEM/Conv	569	0	49.0	67.0	63.0	80.0	100	25.0	31.0	0
	VC/VP	569	0	22.0	45.0	46.2	70.0	100	28.1	48.0	0
	all	1138	0	32.0	58.0	54.6	77.8	100	27.9	45.8	26
NASA_TLX_Frustration	ROTEM/Conv	569	0	23.0	50.0	46.5	68.0	100	27.7	45.0	0
	VC/VP	568	0	10.0	28.5	30.5	48.2	100	23.1	38.2	1
	all	1137	0	18.0	36.0	38.5	60.0	100	26.7	42.0	27

Table 9: Descriptive table - workload scores: joint data set, separated by technology

2 Validation of NASA-TLX

We validate the NASA-TLX score and the different subscores by fitting mixed linear regression models with a random intercept per person (to cover the repeated measurements) and a random intercept per study. We fit univariate models with the following covariates:

- Performance: correct (binary)
- Confidence: confidence category
- Distraction
- Central monitor
- Center
- Gender
- Job
- Daytime (binary: above or below median)
- Playback sequence (binary: first two or later)

2.1 NASA-TLX

	Coefficient	CI lower	CI upper	p-value
Correct: yes	-12.58	-14.52	-10.63	< 0.0001
Confidence: Above median	-18.6	-20.7	-16.51	< 0.0001
Distraction: Yes	9.83	5.69	14.1	< 0.0001
CM: Yes	3.36	-0.22	7.14	0.07
Gender: Female	2.67	-1.94	7.37	0.26
Job: Staff Phys	-8.02	-12.84	-3.29	0.0012
Job: Nurse	-0.73	-7.17	6.22	0.83
Center: UKF	-8.03	-14.58	-2.09	0.01
Center: KSW	6.51	0.87	12.76	0.03
Daytime: Above median	-0.42	-4.81	4.09	0.85
Playback Sequence: 3 or later	-5.91	-8.15	-3.71	< 0.0001

Table 10: Results from the univariate models for NASA-TLX

	Coefficient	CI lower	CI upper	p-value
Correct: yes	-14.43	-16.94	-11.91	< 0.0001
Confidence: Above median	-26.36	-28.98	-23.76	< 0.0001
Gender: Female	5.92	-1.35	13.2	0.12
Job: Staff Phys	-13.59	-20.04	-7.16	0.00012
Center: UKF	-5.07	-12.17	2.04	0.17
Daytime: Above median	0.94	-5.91	7.88	0.79
Playback Sequence: 3 or later	-3.86	-7.23	-0.49	0.03

Table 11: Results from the univariate models for NASA-TLX: data set VC

	Coefficient	CI lower	CI upper	p-value
Correct: yes	-2.73	-8.48	3.02	0.35
Confidence: Above median	-0.42	-6.35	5.51	0.89
Gender: Female	-2.34	-8.07	3.4	0.43
Job: Staff Phys	0.72	-7	8.44	0.86
Job: Nurse	-0.21	-7.81	7.4	0.96
Center: KSW	1.98	-3.74	7.71	0.50
Playback Sequence: 3 or later	-25.67	-29	-22.35	< 0.0001

Table 12: Results from the univariate models for NASA-TLX: data set VP1

	Coefficient	CI lower	CI upper	p-value
Correct: yes	-12.95	-16.24	-9.66	< 0.0001
Confidence: Above median	-12.33	-15.89	-8.8	< 0.0001
Distraction: Yes	9.69	6.2	13.19	< 0.0001
CM: Yes	3.15	-0.01	6.32	0.05
Gender: Female	-1.55	-10.23	7.13	0.73
Job: Staff Phys	-3.31	-14.33	7.71	0.57
Job: Nurse	-0.2	-10.39	10	0.97
Center: KSW	5	-3.66	13.66	0.27
Playback Sequence: 3 or later	-0.27	-3.46	2.92	0.87

Table 13: Results from the univariate models for NASA-TLX: data set VP2

2.2 Mental demand

	Coefficient	CI lower	CI upper	p-value
Correct: yes	-12.99	-15.33	-10.66	< 0.0001
Confidence: Above median	-20.59	-23.09	-18.1	< 0.0001
Distraction: Yes	9.52	4.55	14.54	0.0002
CM: Yes	4.32	0.01	8.7	0.05
Gender: Female	0.91	-4.01	5.92	0.72
Job: Staff Phys	-7.13	-12.24	-1.98	0.007
Job: Nurse	1.3	-5.73	8.71	0.72
Center: UKF	-7.54	-14.41	-1.2	0.02
Center: KSW	8.86	2.65	15.53	0.007
Daytime: Above median	0.67	-4.08	5.53	0.78
Playback Sequence: 3 or later	-1.23	-3.91	1.42	0.37

Table 14: Results from the univariate models for NASA-TLX Mental Demand

2.3 Temporal demand

	Coefficient	CI lower	CI upper	p-value
Correct: yes	-10.53	-12.8	-8.27	< 0.0001
Confidence: Above median	-15.97	-18.48	-13.47	< 0.0001
Distraction: Yes	8.33	3.59	13.08	0.0006
CM: Yes	0.77	-3.34	4.91	0.72
Gender: Female	4.83	-1.87	11.69	0.16
Job: Staff Phys	-11.07	-18.06	-4.2	0.0021
Job: Nurse	-3.98	-13.37	6.01	0.41
Center: UKF	-14.36	-24.02	-5.75	0.0019
Center: KSW	9.95	2.12	18.64	0.02
Daytime: Above median	-0.03	-6.1	6.42	0.99
Playback Sequence: 3 or later	-3.24	-5.78	-0.74	0.01

Table 15: Results from the univariate models for NASA-TLX Temporal Demand

2.4 Reverse NASA-TLX overall

	Coefficient	CI lower	CI upper	p-value
Correct: yes	-18.11	-20.7	-15.52	< 0.0001
Confidence: Above median	-29.49	-32.2	-26.82	< 0.0001
Distraction: Yes	12.5	6.93	18.32	< 0.0001
CM: Yes	2.04	-2.76	7.2	0.42
Gender: Female	4.59	-0.56	9.89	0.09
Job: Staff Phys	-8.41	-13.96	-3	0.0031
Job: Nurse	-2.22	-9.62	5.94	0.57
Center: UKF	-4.86	-12.7	1.62	0.16
Center: KSW	6.31	-0.1	14.4	0.07
Daytime: Above median	-0.11	-5.16	5.12	0.97
Playback Sequence: 3 or later	-2.45	-5.55	0.53	0.11

Table 16: Results from the univariate models for Reverse NASA-TLX Overall

2.5 Effort

	Coefficient	CI lower	CI upper	p-value
Correct: yes	-12.99	-15.43	-10.57	< 0.0001
Confidence: Above median	-19.69	-22.32	-17.07	< 0.0001
Distraction: Yes	9.8	4.65	15	0.00022
CM: Yes	5.89	1.43	10.41	0.01
Gender: Female	1.61	-3.53	6.85	0.54
Job: Staff Phys	-7.6	-12.94	-2.23	0.006
Job: Nurse	1.19	-6.14	9.01	0.75
Center: UKF	-7.72	-15.19	-1.05	0.03
Center: KSW	9.07	2.59	16.24	0.0085
Daytime: Above median	-0.04	-4.99	5.05	0.99
Playback Sequence: 3 or later	-0.7	-3.48	2.03	0.62

Table 17: Results from the univariate models for NASA-TLX Effort

2.6 Frustration

	Coefficient	CI lower	CI upper	p-value
Correct: yes	-14.21	-16.58	-11.85	< 0.0001
Confidence: Above median	-21.44	-23.99	-18.89	< 0.0001
Distraction: Yes	8.57	3.41	13.59	0.00098
CM: Yes	3.1	-1.43	7.43	0.17
Gender: Female	1.16	-5.46	7.81	0.73
Job: Staff Phys	-6.93	-13.68	0.01	0.05
Job: Nurse	5.19	-4.19	14.7	0.28
Center: UKF	-9.07	-17.98	-0.19	0.05
Center: KSW	0.99	-7.21	9.2	0.81
Daytime: Above median	-1.94	-7.97	4.37	0.54
Playback Sequence: 3 or later	-0.17	-2.87	2.53	0.90

Table 18: Results from the univariate models for NASA-TLX Frustration

3 Comparison of technologies

To explore more in depth why the NASA-TLX and its subscores were better in the case of the new visual technologies, we looked at two different things:

- To see which subscore profited the most from the new techologies, we calculated univariate models for each subscore that included only the technology variable, and we compared the size of the estimated coefficients.
- To characterize the individuals who profited the most from the introduction of the new technologies, we fitted a joint model for the total NASA-TLX with the technology variable and several other covariates. In one additional model per variable, we included an interaction term between technology and the respective covariate to see if the impact of certain variables was particularly strong in the case of the new technologies.

3.1 Comparison of subscores

It seems that the impact of the new technologies is very comparable between the subscales. The largest coefficient is found in the effort subscale, but all coefficients are quite close to each other.

	Coefficient	CI lower	CI upper	p-value
Total	-14.36	-16.06	-12.65	< 0.0001
Mental demand	-15.79	-17.85	-13.72	< 0.0001
Temporal demand	-10.53	-12.59	-8.46	< 0.0001
Effort	-16.8	-18.92	-14.68	< 0.0001
Frustration	-15.97	-18.06	-13.87	< 0.0001
Reverse NASA-TLX overall	-18.28	-20.63	-15.93	< 0.0001

Table 19: Coefficients for the comparison of VC/VP with conventional technologies for different NASA-TLX subscores

3.2 Interaction terms

As a start, we looked at interactions between technology and sex, age group and job, respectively. As none of these interactions was significant, we conclude that none of the respective variables is especially responsible for the lower workload in the new technologies.

4 Open questions

The following open questions remain:

- Which additional variables could be of interest in the last section?

R version and packages used to generate this report

R version: R version 3.6.2 (2019-12-12)
Base packages: stats, graphics, grDevices, utils, datasets, methods, base
Other packages: lmerTest, lme4, Matrix, dplyr, stringr, reporttools, xtable, ggplot2, knitr
This document was generated on 2020-04-14 at 12:11.
